# Pemphigus Vulgaris Mimicking Seborrheic Dermatitis: A Case Report

**DOI:** 10.7759/cureus.71357

**Published:** 2024-10-13

**Authors:** Brigitte L Cochran, Jennifer Jallo, Leah Taylor, Harold Essenfeld

**Affiliations:** 1 Osteopathic Medicine, Philadelphia College of Osteopathic Medicine, Moultrie, USA; 2 Family Medicine, Lincoln Memorial University-DeBusk College of Osteopathic Medicine, Harrogate, USA; 3 Dermatology, Total Dermatology Care Center, Jacksonville, USA; 4 Dermatopathology, Institute for Immunofluorescence, Pompano Beach, USA

**Keywords:** clinical dermatology, -dermatopathology, h&e staining, pemphigus vulgaris, seborrheic dermatitis

## Abstract

This case discusses a 55-year-old patient who was evaluated at a dermatology outpatient clinic for a chronic, pruritic, and painful hyperkeratotic plaque located on her scalp vertex. Given the size of the plaque and its thick, yellow scales, an initial clinical diagnosis of seborrheic dermatitis was favored. However, after weeks of unsuccessful treatment with ketoconazole shampoo, topical fluocinolone 0.01% oil, mupirocin ointment, and oral doxycycline, it became apparent that deeper investigation and reconsideration of the original diagnosis were warranted. A punch biopsy and direct immunofluorescence of the lesion were performed, and the results revealed a final diagnosis of pemphigus vulgaris (PV). This case highlights the complexity of dermatology and the challenges in achieving an accurate diagnosis based solely on clinical features, mainly when diseases exhibit overlapping characteristics or are present in less familiar ways. Distinguishing between conditions with similar features can be particularly difficult and becomes even more crucial when one condition is benign, and another is potentially fatal. Therefore, we recommend further investigating when a skin condition does not resolve or fails to respond to multiple treatment attempts.

## Introduction

Pemphigus describes a group of rare autoimmune dermatoses that affect the epithelium of mucocutaneous membranes, resulting in persistent acantholysis [1]. This autoimmune reaction disrupts the protein connections between epithelial cells of the skin and mucous membranes, forming loose blisters that easily separate the upper and lower skin layers upon friction. Patients often present with generalized bullae and erosions, which can lead to serious and fatal complications such as infections, severe dehydration, and protein loss [2].

Among the many types, pemphigus vulgaris (PV) is the most common type of pemphigus disorder and has an unclear pathogenesis [3]. However, several studies have associated PV with specific autoantibodies that target cadherins, proteins crucial for binding skin cells together. This targeting causes acantholysis and the separation of keratinocytes [4]. Environmental factors, such as diet, sleep, stress, medications, viral infections, and ultraviolet radiation, may contribute to flares of PV by exacerbating immune dysregulation [3,5]. 

Oropharyngeal involvement is observed in over 90% of patients with PV, with the oral mucosa often being the initial site of disease manifestation [6]. PV with scalp involvement is relatively uncommon, and cases of PV solely localized to the scalp are even rarer, with fewer than 30 documented patient cases in the literature [1]. When PV presents as localized scalp involvement, it typically manifests as a combination of ulcers, erosions, and plaques [7]. It has been reported that PV involving the scalp in a majority of patients can potentially lead to future alopecia. This underscores the importance of effective treatment to manage the disease and mitigate the risk of further complications [7-10]. 

In comparison, seborrheic dermatitis (SD) is an inflammatory skin reaction to the presence of Malassezia yeast, which is part of the skin’s natural flora. It is a chronic, recurring skin condition characterized by erythematous and pruritic patches or plaques with varying degrees of scaling [11]. SD typically affects areas rich in sebaceous glands, such as the scalp, ears, nasolabial folds, glabella, and superior cutaneous eyebrows. SD often forms plaques on the scalp. Treatment for SD aims to reduce cutaneous colonization of Malassezia, reduce inflammation, and regulate sebum production [12]. 

We present a case report of a patient with a focal plaque on the scalp vertex that was clinically perceived as SD. However, biopsy results later confirmed the diagnosis of PV.

## Case presentation

**Figure 1:**
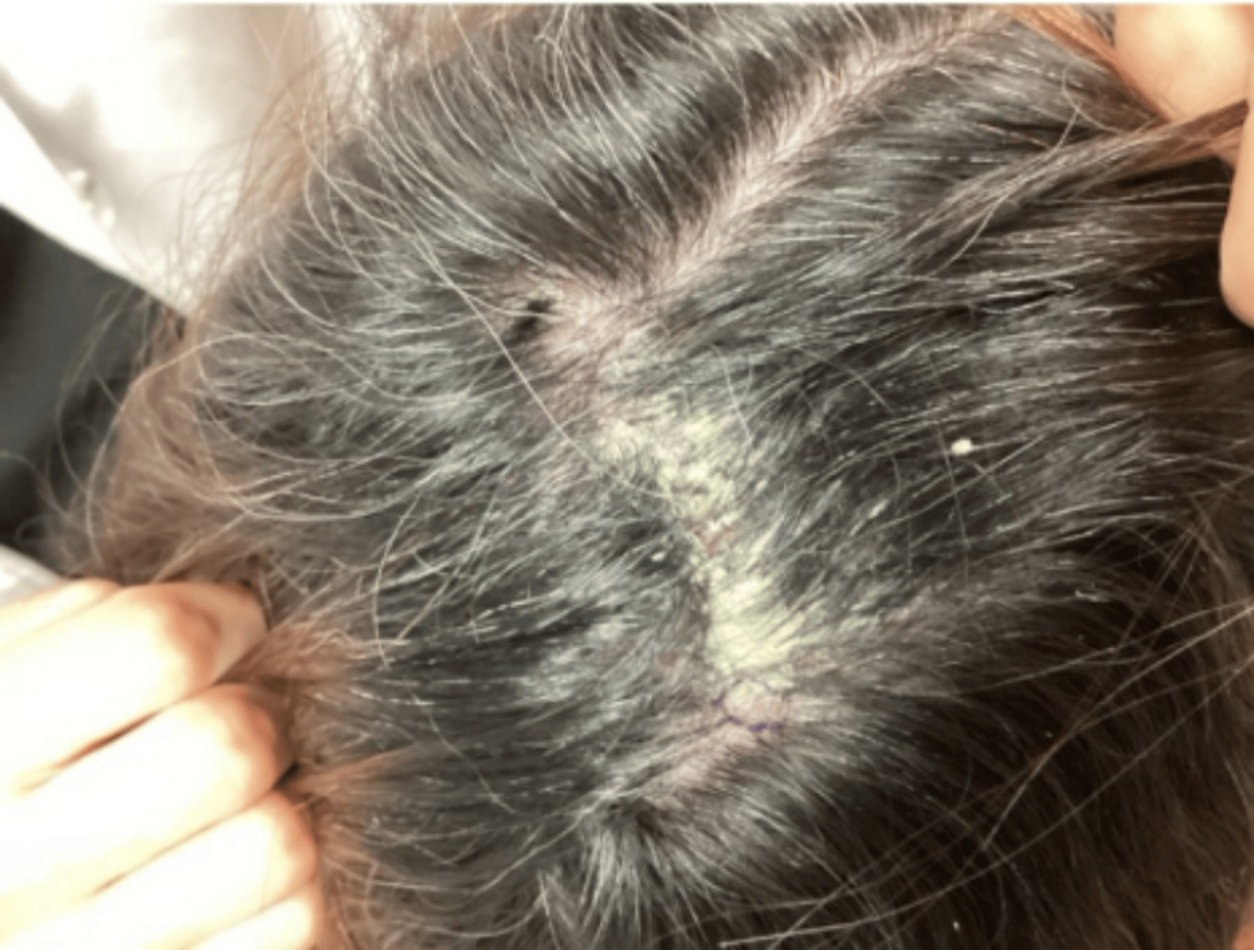
Gross clinical presentation of a sebum-colored plaque located on the scalp vertex at the initial office visit.

A 55-year-old woman presented to the dermatology outpatient clinic with a tender, pruritic, hyperkeratotic plaque on the vertex of her scalp that had been present for four months. Initially diagnosed as SD, the lesion had been treated with a two-week course of oral doxycycline, topical fluocinolone 0.01% oil, ketoconazole shampoo, and topical mupirocin 2% ointment. On examination, the plaque measured 6 cm by 4 cm, exhibited a thick, yellow scale adherent to the proximal hair shaft, and was accompanied by at least three smaller surrounding hyperkeratotic plaques (Figure 1). The patient reported no associated hair loss, lymphadenopathy, fever, or malaise. A punch biopsy from the right superior parietal scalp was performed for hematoxylin and eosin (H&E) staining, which suggested pemphigus foliaceus. The gross clinical presentation of a sebum-colored plaque located on the scalp vertex during the initial office visit is shown in Figure 1. Differential diagnosis also included psoriasis, discoid lupus erythematosus, Brunsting-Perry pemphigoid, and sarcoidosis. 

**Figure 2:**
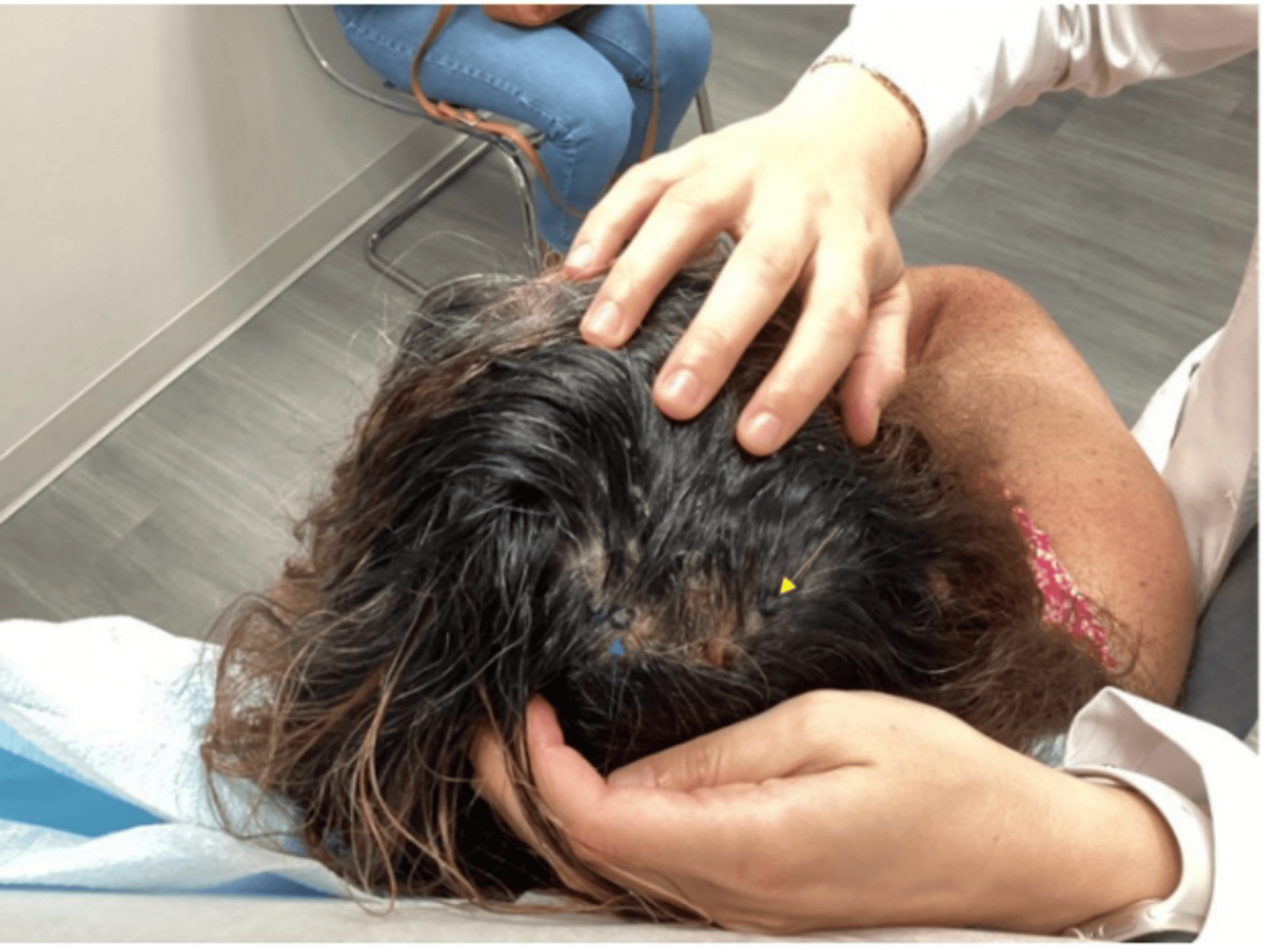
Biopsy sites marked for punch biopsy for H&E staining of the scalp vertex (left circled site, yellow arrow) and DIF of the right scalp (right circled site, blue arrow) during the follow-up visit after no improvement. H&E: hematoxylin and eosin; DIF: direct immunofluorescence.

**Figure 3:**
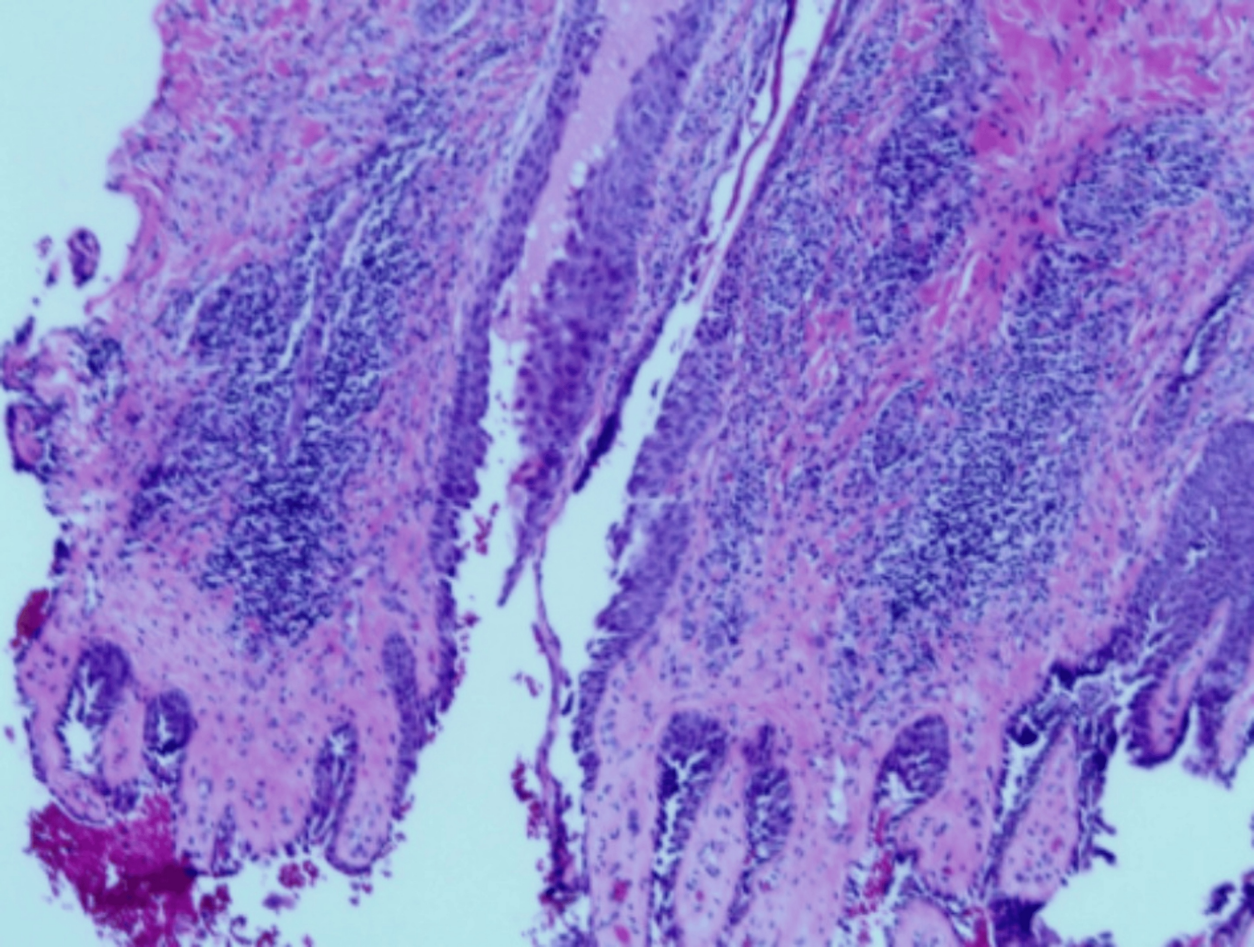
Acantholysis hair follicle on H&E staining of the central scalp vertex (magnification: 20x). H&E: hematoxylin and eosin.

**Figure 4:**
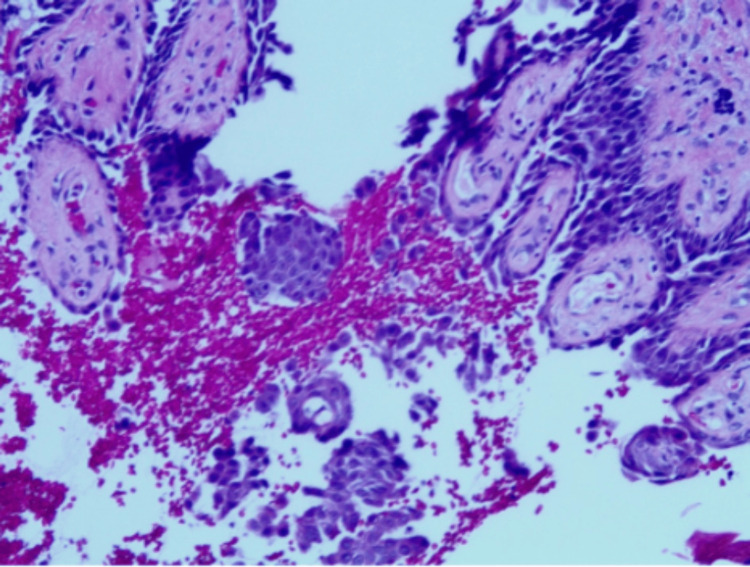
Suprabasilar intraepidermal acantholytic blister with involvement of upper epithelial adnexae and prominent eosinophilic spongiosis on H&E staining of the central scalp vertex. There are no acari/ectoparasite elements, interface/lichenoid pattern, or herpetic viral cytopathic changes identified in examined slides. The PAS stain was negative for microorganisms. These histologic findings strongly support PV over pemphigus foliaceus or other variants (magnification: 40x). H&E: hematoxylin and eosin; PV: pemphigus vulgaris; PAS: periodic acid-Schiff.

**Figure 5:**
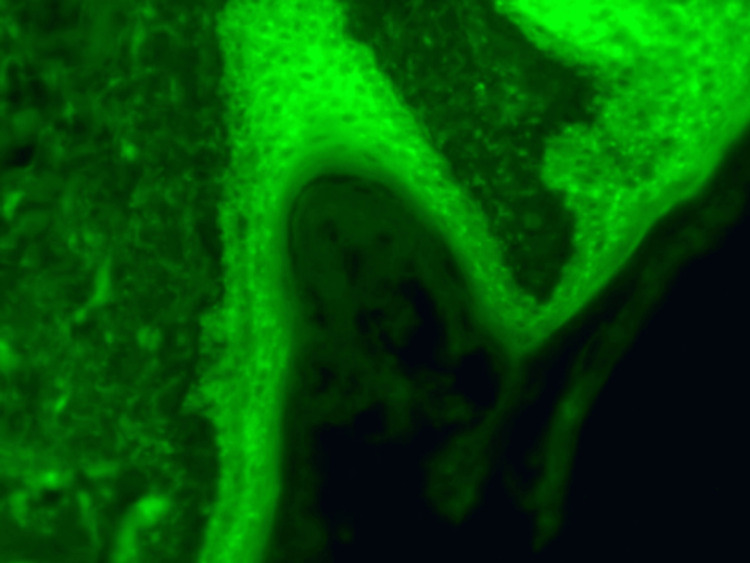
DIF stain demonstrated linear/granular IgG and IgG4 deposition throughout the epithelial cell surfaces. There are also linear/granular C3 deposits on the lower two-thirds of the epithelial strata. There are no immunoreactants at the basement membrane zone, and no IgA, IgM, C5b-9, or fibrinogen deposits are seen in this specimen. These immunofindings are diagnostic for pemphigus, and this immunofluorescence pattern supports the diagnosis of PV over pemphigus foliaceus or other variants. DIF: direct immunofluorescence; PV: pemphigus vulgaris.

However, in the following weeks, the patient did not show improvement with the current treatment regimen. Consequently, direct immunofluorescence was recommended for further evaluation, along with a second H&E stain of the right scalp and scalp vertex (Figures 2-5).

After the biopsy confirmed a diagnosis of PV, the patient was subsequently treated with an oral prednisone taper: 30 mg for one week, then decreasing by 5 mg per week for a total of six weeks. The patient states that the appearance of the lesion remained unchanged after completing the steroid taper; however, she reported improvement in pain in the affected areas. In another follow-up visit, the patient was treated with an intralesional triamcinolone injection in the affected area. 

## Discussion

PV is a disease characterized by cycles of remission and flare-ups throughout a patient's lifetime. Treatment approaches vary depending on disease severity. Cases presenting with localized scalp involvement, such as in this case report, are uncommon; however, treatment is still indicated to reduce the risk of secondary infection, reduce pain, and improve quality of life. 

Treatment generally often relies on systemic immunosuppressive agents and high-potency topical steroids, which can effectively manage disease and control flares [13]. In this case, as the patient was only prescribed a high-potency topical steroid, it is crucial to educate patients on the appropriate use of topical steroids, as it should only be used for flares after initial remission to avoid complications such as steroid atrophy from prolonged use [14].

More severe cases of PV involving mucous membranes have untreated mortality rates reaching as high as 50%, with the main cause of death being septicemia. However, with appropriate treatment, the mortality rate drops to about 10% [14]. Common treatments for mucocutaneous or mucosal PV include systemic corticosteroids and immunosuppressive agents, such as rituximab [14]. Other agents, including azathioprine and mycophenolate mofetil, are sometimes used alongside systemic corticosteroids to minimize long-term steroid use and its associated adverse effects [1]. In the case of refractory disease, further interventions such as intravenous immunoglobulin (IVIG), immunoadsorption, and cyclophosphamide may be considered [1]. 

PV is a chronic disease requiring frequent follow-ups and close medical surveillance to make medication changes and dose adjustments based on the patient’s response to treatment. PV localized to the scalp is very rare, with less than 30 case reports published citing this presentation of the disease [13]. Clinicians should routinely screen all patients with localized scalp PV for mucocutaneous involvement. 

Due to the high prevalence of skin disorders that can mimic each other and/or malignancies, it is important for the clinician to be aware of less common presentations of skin lesions. Clinicians should have a small window of topical and/or systemic treatment before biopsy is performed to rule in the final diagnosis for optimal treatment. 

## Conclusions

We present an unusual case in which the presentation of PV in the scalp mimicked SD due to the exudate forming an adherent scale/crust on the hair shafts. Due to the differing nature of these conditions and their potential complications, it is important for clinicians to recognize that either could mimic the other.

This case is particularly noteworthy because PV presented exclusively on the scalp vertex, an exceptionally uncommon localization for this disease. Therefore, if initial treatment for SD, such as zinc pyrithione and/or ketoconazole, does not show improvement, a biopsy should be considered to determine the final diagnosis and guide effective treatment. Awareness of such atypical presentations is essential for ensuring proper diagnosis and management.
